# Damage control operations in non-trauma patients: defining criteria for the staged rapid source control laparotomy in emergency general surgery

**DOI:** 10.1186/s13017-016-0067-4

**Published:** 2016-02-24

**Authors:** Robert D. Becher, Andrew B. Peitzman, Jason L. Sperry, Jared R. Gallaher, Lucas P. Neff, Yankai Sun, Preston R. Miller, Michael C. Chang

**Affiliations:** Section of General Surgery, Trauma, and Surgical Critical Care, Department of Surgery, Yale School of Medicine, 330 Cedar Street, BB 310, New Haven, CT 06510 USA; Department of Surgery, University of Pittsburgh School of Medicine, Pittsburgh, PA USA; Department of Surgery, University of North Carolina School of Medicine, Chapel Hill, NC USA; Department of Surgery, David Grant Medical Center, Travis Air Force Base, Fairfield, CA USA; Department of Surgery, Wake Forest University School of Medicine, Winston-Salem, NC USA

**Keywords:** Damage control, Emergency general surgery, EGS, Rapid source control laparotomy, Physiologically decompensated

## Abstract

**Background:**

The staged laparotomy in the operative management of emergency general surgery (EGS) patients is an extension of trauma surgeons operating on this population. Indications for its application, however, are not well defined, and are currently based on the lethal triad used in physiologically-decompensated trauma patients. This study sought to determine the acute indications for the staged, rapid source control laparotomy (RSCL) in EGS patients.

**Methods:**

All EGS patients undergoing emergent staged RSCL and non-RSCL over 3 years were studied. Demographics, physiologic parameters, perioperative variables, outcomes, and survival were compared. Logistic regression models determined the influence of physiologic parameters on mortality and postoperative complications. EGS-RSCL indications were defined.

**Results:**

215 EGS patients underwent emergent laparotomy; 53 (25 %) were staged RSCL. In the 53 patients who underwent a staged RSCL based on the lethal triad, adjusted multivariable regression analysis shows that when used alone, no component of the lethal triad independently improved survival. Staged RSCL may decrease mortality in patients with preoperative severe sepsis / septic shock, and an elevated lactate (≥3); acidosis (pH ≤ 7.25); elderly (≥70); male gender; and multiple comorbidities (≥3). Of the 162 non-RSCL emergent laparotomies, 27 (17 %) required unplanned re-explorations; of these, 17 (63 %) had sepsis preoperatively and 9 (33 %) died.

**Conclusions:**

The acute physiologic indicators that help guide operative decisions in trauma may not confer a similar survival advantage in EGS. To replace the lethal triad, criteria for application of the staged RSCL in EGS need to be defined. Based on these results, the indications should include severe sepsis / septic shock, lactate, acidosis, gender, age, and pre-existing comorbidities. When correctly applied, the staged RSCL may help to improve survival in decompensated EGS patients.

## Background

The staged laparotomy in the operative management of select trauma patients is designed to ensure their immediate survival [1–6]. The damage control (DC) laparotomy is therefore not an operation of last resort; rather, it is a well thought-out stage on a continuum of care which prioritizes the restoration of physiologic normality and homeostasis above definitive organ repair and anatomic reconstruction.

Application of DC principles are based on the clinical recognition of a trauma patient who is physiologically decompensated as defined by the lethal triad seen with hemorrhagic shock: acidosis, coagulopathy, and hypothermia. Decompensated trauma patients must be rescued to avoid progression to irreversible physiologic exhaustion and death; abbreviated operations allow stabilization, correction, and reevaluation of physiologic derangements in an intensive care unit setting [6–13].

Similarly, a select group of emergency general surgery (EGS) patients also present decompensated, near irreversible physiologic exhaustion and death, but for different reasons, often driven by severe sepsis or septic shock [14]. As a natural extension of trauma surgeons operating on the EGS patient-population, DC principles have been widely applied to the operative management of EGS patients.

Indications for DC application in EGS patients, however, are not well defined, and are currently based on the lethal triad used in trauma patients. Because the lethal triad in trauma is founded on patients presenting with hemorrhagic shock – something less commonly seen in EGS patients – its use to guide operative decision making in EGS patients may not be appropriate.

This study sought to determine the acute physiologic indications for the staged, rapid source control laparotomy (RSCL) in EGS patients. Our hypothesis was that application of the lethal triad to EGS patients as criteria for implementation of abbreviated laparotomy would not improve outcomes, and that the definition of a physiologically decompensated EGS patient would be unique from that used in trauma patients.

## Methods

This is a retrospective review of consecutive EGS patients undergoing emergent, non-trauma related laparotomies over 3 years, from March 1, 2007 to March 1, 2010, at a tertiary medical center. Emergent operations were defined in accordance with the National Surgical Quality Improvement Program’s (NSQIP) definition of an emergency case: one performed as soon as possible and no later than 12 h after the patient has been admitted to the hospital or after the onset of related preoperative symptomatology [15].

Variables were collected through electronic chart review. Data included demographics, physiologic parameters, perioperative variables, outcomes, and survival. Magnitude of the inflammatory response (four categories: no inflammation, SIRS, sepsis, or severe sepsis/septic shock) was classified based on the NSQIP definition; this definition is in concert with standardized definitions outlined by the American College of Chest Physicians (ACCP), the Society of Critical Care Medicine (SCCM), and the Surgical Infection Society (SIS) [16].

Patients were identified as undergoing a rapid source control laparotomy (RSCL; i.e., damage control laparotomy; planned re-laparotomy) if there was a deliberate, preemptive plan to perform an immediate, limited, staged operation as part of an ongoing continuum of resuscitation. The decision to proceed with staged RSCL was based on the lethal triad used in trauma patients; such RSCL intentions were identified in the operative note, which had to address the planned attempt to reverse the pre-terminal effects of pre-operative physiologic derangements, and/or intra-abdominal hypertension, by means of an abbreviated laparotomy. These patients were left with an open abdomen with a negative pressure dressing at the end of the initial operation, and transferred to the intensive care unit for stabilization. The second operation was then governed by a planned re-laparotomy, once resuscitation and stabilization had been achieved.

Non-RSCL patients were those who underwent non-staged operations, and had their fascia closed at the completion of the initial operation. If these patients subsequently required a re-laparotomy, this would be considered an unplanned re-laparotomy, or an “on-demand re-laparotomy” [17, 18]. For the current analyses, only patients who underwent open operations with colon or small bowel resections were included, based on their high degree of postoperative morbidity and mortality, comparable source for sepsis, and the increased power of the analyses [19–21].

Patient characteristics, physiologic parameters, perioperative variables, outcome metrics, and survival were first compared using univariate techniques. Chi-squared analysis (*χ*2) or Fisher’s exact test were used to compare differences in proportions of categorical variables; such data were summarized by percentages. Student’s t-tests were used to compare normally distributed continuous variables; such data were summarized by mean values with standard deviations (±SD). Wilcoxon rank sum tests were used to compare non-normally distributed continuous variables; such data were summarized by median values with interquartile ranges.

In hospital mortality was the primary outcome measure. Logistic regression models determined the influence of acute physiologic parameters and other patient characteristics on in-hospital mortality; analyses assessing determinants of postoperative complications were additionally completed. All candidate covariates for multivariable regression were included in a full selection model; variables were not excluded based on their statistical significance. Significance was defined as *p*-value < 0.05 throughout. EGS-based RSCL indications were defined.

All statistical analyses were conducting using a commercially available statistics software program (SAS 9.2; SAS Institute Inc., Cary, NC). Approval was obtained from the Wake Forest University Baptist Medical Center Institutional Review Board.

## Results

215 EGS patients underwent emergent laparotomy during the study period, 162 (75 %) were non-rapid source control laparotomy (non-RSCL) operations and 53 (25 %) were staged rapid source control laparotomy (RSCL; damage control; planned re-laparotomy). Comparing the two operative groups, there were no significant differences in patient characteristics, including gender, race, age, or comorbidities (Table 1).

**Table 1:** Patient characteristics and peri-operative physiologic scores & metrics, by operation type

Variable^a^	non-RSCL (*n* = 162)	RSCL (*n* = 53)	*p*-value^b^
Male gender	94 (58.0 %)	29 (54.7 %)	0.6727
Race & Ethnicity:			
White	128 (79.0 %)	46 (86.8 %)	0.2107
Black	27 (16.7 %)	6 (11.2 %)	0.3486
Age, years	61 (17)	64 (13)	0.2336
Age (≥70)	55 (34.0 %)	19 (35.9 %)	0.8006
Comorbidities:			
Number comorbidities, average	1.9 (2.2)	2.2 (2.8)	0.4641
No comorbidities	71 (43.8 %)	23 (43.4 %)	0.9562
1 comorbidity	19 (11.7 %)	2 (3.8 %)	0.0904
2 comorbidities	14 (8.6 %)	7 (13.2 %)	0.3311
≥3 comorbidities	58 (35.8 %)	21 (39.6 %)	0.6166
Inflammatory status preoperatively:			
No inflammation	75 (46.3 %)	10 (18.9 %)	0.0004
Sepsis	59 (36.4 %)	28 (52.8 %)	0.0346
Severe sepsis/septic shock	28 (17.3 %)	15 (28.3 %)	0.0817
Lethal Triad:			
Hypothermia (temp ≤ 35C)	24 (16.8 %)	7 (14.9 %)	0.7610
Coagulopathy (INR ≥ 1.7)	13 (11.1 %)	9 (18.4 %)	0.2085
Acidosis (pH ≤ 7.25)	24 (22.2 %)	18 (35.3 %)	0.0810
Patient characteristics:			
ASA score, average	3 (1)	4 (1)	<0.0001
ASA score, 1 or 2	23 (14.2 %)	0 (0.0 %)	0.0015
ASA score, 3	65 (40.1 %)	8 (15.1 %)	0.0008
ASA score, 4 or 5	74 (45.7 %)	45 (84.9 %)	<0.0001
Lactate (≥3)	32 (33.3 %)	26 (51.0 %)	0.0372

*RSCL* rapid source control laparotomy, *PRBC* packed red blood cells, *C* Celsius, *INR* international normalized ratio, *ASA* American Society of Anesthesiologists, *L* liters

^a^Categorical variables are presented as number (%); continuous data are presented as mean (± standard deviation); and non-normally distributed continuous variables as median (interquartile range)

^b^ *P*-values for overall tests of differences between the groups from *χ*2 or Fisher’s Exact Test for categorical variables and one-way analysis of variance or Kruskal-Wallis test for continuous variables; *p*-value of <0.05 significant

Peri-operative physiologic scores and metrics, however, did significantly differ (Table 1). Compared with emergent patients in the RSCL cohort, the non-RSCL group was overall healthier, with a significantly higher percentage of patients with no preoperative inflammation and with lower ASA scores (1, 2, or 3). The two groups did not differ significantly with regard to patients with severe sepsis / septic shock, hypothermia, coagulopathy, or acidosis. No patients in either group fit only into the SIRS only classification of preoperative inflammation, indicating that all had a presumed source of infection.

Overall mortality in the RSCL group was significantly higher than in the non-RSCL population (45 % vs 20 %) (Table 2). Specifically, mortality was higher in RSCL compared to non-RSCL patients who were female, white, and without comorbidities. Interestingly, however, mortality between the two operative approaches was no different for patients with sepsis or severe sepsis / septic shock; males; elderly (age ≥70); patients with multiple comorbidities (≥3); patients with hypothermia (temperature ≤35C), coagulopathy (INR ≥1.7), and acidosis (pH ≤ 7.25) (the lethal triad criteria); for all ASA scoring levels; and for elevated lactate levels.

**Table 2:** Rates of in-hospital mortality for patient characteristics and peri-operative physiologic metrics, by operation type

Variable^a^	non-RSCL (*n* = 162)	RSCL (*n* = 53)	*p*-value^b^
All patients:	33 (20.4 %)	24 (45.3 %)	0.0004
Inflammatory status preoperatively:			
No inflammation	8 (10.7 %)	5 (50.0 %)	0.0012
Sepsis	10 (19.9 %)	10 (35.7 %)	0.0520
Severe sepsis/septic shock	15 (53.6 %)	9 (60.0 %)	0.6858
Patient Characteristics:			
Male Gender	25 (26.6 %)	13 (44.8 %)	0.0632
Female Gender	8 (11.7 %)	11 (45.8 %)	0.0004
Race, White	27 (21.1 %)	20 (43.5 %)	0.0034
Age (≥70)	20 (36.4 %)	10 (52.6 %)	0.2131
No comorbidities	17 (23.9 %)	11 (47.8 %)	0.0295
1 comorbidity	0 (0.0 %)	1 (50.0 %)	0.0016
2 comorbidities	3 (21.4 %)	3 (42.9 %)	0.3055
≥3 comorbidities	13 (22.4 %)	9 (42.9 %)	0.0733
Lethal Triad:			
Hypothermia (temp ≤ 35C)	8 (33.3 %)	3 (42.9 %)	0.6431
Coagulopathy (INR ≥ 1.7)	5 (38.5 %)	5 (55.6 %)	0.4285
Acidosis (pH ≤ 7.25)	12 (50.0 %)	10 (55.6 %)	0.7213
Patient characteristics:			
ASA score, 1 or 2	0 (0.0 %)	0 (0.0 %)	-
ASA score, 3	5 (7.7 %)	1 (12.5 %)	0.6404
ASA score, 4 or 5	28 (37.8 %)	23 (51.1 %)	0.1559
Lactate (≥3)	13 (40.6 %)	11 (42.3 %)	0.8970

*RSCL* rapid source control laparotomy, *PRBC* packed red blood cells, *C* Celsius, *INR* international normalized ratio, *ASA* American Society of Anesthesiologists, *L* liters

^a^Categorical variables are presented as number (%); continuous data are presented as mean (± standard deviation); and non-normally distributed continuous variables as median (interquartile range)

^b^ *P*-values for overall tests of differences between the groups from *χ*2 or Fisher’s Exact Test for categorical variables and one-way analysis of variance or Kruskal-Wallis test for continuous variables; *p*-value of <0.05 significant

The application of the lethal triad to guide the use of RSCL did not discriminate between survivors and non-survivors in our multivariable logistic regression models (Table 3). Patients with hypothermia, coagulopathy, and acidosis who underwent damage control procedures had similar rates of mortality compared with the non-RSCL group.

**Table 3:** Logistic regression analyses predicting in-hospital mortality for RSCL patients, based only on the lethal triad

Variable^a^	Unadjusted model^a^		Adjusted multivariable model^b^	
	OR (95 % CI)	*p*-value^c^	OR (95 % CI)	*p*-value^c^
Lethal Triad:				
Hypothermia (temp ≤ 35C)	0.83 (0.16–4.19)	0.8205	0.70 (0.13–3.74)	0.5330
Coagulopathy (INR ≥ 1.7)	1.53 (0.36–6.55)	0.5680	1.52 (0.34–6.87)	0.5884
Acidosis (pH ≤ 7.25)	1.92 (0.60–6.15)	0.2703	1.70 (0.47–6.09)	0.4167

*RSCL* rapid source control laparotomy, *C* Celsius, *INR* international normalized ratio

^a^Unadjusted univariate analyses were used to test an independent variables’s predictive ability on in-hospital mortality

^b^Model covariates for mortality were chosen by full selection

^c^ *P*-value of <0.05 significant

In emergent operative patients undergoing non-RSCL (Table 4), multiple variables increased the chances of mortality in the univariate analyses, including severe sepsis/septic shock, male gender, elderly, acidosis, elevated lactate, and ASA score of 4 or 5. Two variables in this group portended an increased likelihood of survival: patients without inflammation and those with an ASA score of 3. The adjusted multivariable logistic regression model confirmed that four variables significantly and independently increased the chances of mortality for non-RSCL operations: patients with severe sepsis / septic shock, male gender, elderly, and acidosis.

**Table 4:** Logistic regression analyses predicting in-hospital mortality for non-RSCL patients

Variable^a^	Unadjusted model^a^		Adjusted multivariable model^b^	
	OR (95 % CI)	*p*-value^c^	OR (95 % CI)	*p*-value^c^
Preoperative inflammatory status:				
No inflammation	0.30 (0.12–0.71)	0.0060	1.00	-
Sepsis	0.71 (0.31–1.62)	0.4146	0.18 (0.02–1.95)	0.1583
Severe sepsis/septic shock	7.44 (3.04–18.17)	<0.0001	12.32 (1.19–127.33)	0.0350
Patient Characteristics:				
Male Gender	2.72 (1.14–6.47)	0.0240	23.98 (2.00–287.34)	0.0122
Race, White	1.25 (0.47–3.32)	0.6579	0.25 (0.01–5.64)	0.3857
Age (≥70)	4.13 (1.86–9.19)	0.0005	13.77 (1.30–145.70)	0.0293
No comorbidities	1.48 (0.69–3.18)	0.3201	1.00	-
1 comorbidity	0.001 (0.001–999.99)	0.9546	0.001 (0.001–999.99)	0.9132
2 comorbidities	1.07 (0.284.09)	0.9181	0.04 (0.01–4.13)	0.1746
≥3 comorbidities	1.21 (0.55–2.66)	0.6299	0.07 (0.01–0.57)	0.0139
Preoperative metrics:				
Acidosis (pH ≤ 7.25)	3.94 (1.51–10.30)	0.0052	11.08 (1.02–120.64)	0.0484
Lactate (≥3)	2.68 (1.06–6.82)	0.0378	0.62 (0.08–4.78)	0.6425
Coagulopathy (INR ≥ 1.7)	2.08 (0.62–6.97)	0.2333	2.11 (0.22–20.43)	0.5183
ASA score, 1 or 2	0.001 (0.001–999.99)	0.9672	1.00	-
ASA score, 3	0.21 (0.08–0.57)	0.0022	999 (0.001–999.99)	0.9574
ASA score, 4 or 5	10.10 (3.65–27.95)	<0.0001	999 (0.001–999.99)	0.9416

*RSCL* rapid source control laparotomy, *PRBC* packed red blood cells, *C* Celsius, *INR* international normalized ratio, *ASA* American Society of Anesthesiologists

^a^Unadjusted univariate analyses were used to test an independent variables’s predictive ability on in-hospital mortality

^b^Model covariates for mortality were chosen by full selection

^c^ *P*-value of <0.05 significant

In contrast to the non-RSCL group, there were no variables in the unadjusted univariate analyses for the RSCL cohort (Table 5) that increased the odds of mortality. In the adjusted multivariable regression, patients had significantly improved chances of survival when undergoing RSCL with two existing factors: elevated preoperative lactate levels or multiple comorbidities. Patients with preoperative acidosis had significant increased risk of death with RSCL, just as in the non-RSCL group.

**Table 5:** Logistic regression analyses predicting in-hospital mortality for RSCL patients

Variable^a^	Unadjusted model^a^		Adjusted multivariable model^b^	
	OR (95 % CI)	*p*-value^c^	OR (95 % CI)	*p*-value^c^
Preoperative inflammatory status:				
No inflammation	1.26 (0.32–5.01)	0.7397	1.00	-
Sepsis	0.44 (0.15–1.32)	0.1415	0.60 (0.07–5.39)	0.6446
Severe sepsis/septic shock	2.30 (0.68–7.80)	0.1811	3.29 (0.26–41.92)	0.3596
Patient Characteristics:				
Male Gender	0.96 (0.32–2.85)	0.9416	0.55 (0.07–4.28)	0.5708
Race, White	0.58 (0.12–2.88)	0.5022	0.26 (0.01–8.47)	0.4509
Age (≥70)	1.59 (0.51–4.92)	0.4230	5.19 (0.75–35.74)	0.0942
No comorbidities	1.20 (0.40–3.57)	0.7448	1.00	-
1 comorbidity	1.22 (0.07–20.55)	0.8915	999 (0.001–999.99)	0.9848
2 comorbidities	0.89 (0.18–4.45)	0.8900	0.58 (0.05–6.08)	0.6458
≥3 comorbidities	0.85 (0.28–2.58)	0.7739	0.06 (0.01–0.70)	0.0252
Preoperative metrics:				
Acidosis (pH ≤ 7.25)	1.92 (0.60–6.15)	0.2703	19.42 (1.74–216.65)	0.0159
Lactate (≥3)	0.79 (0.26–2.40)	0.6831	0.10 (0.01–0.93)	0.0431
Coagulopathy (INR ≥ 1.7)	1.53 (0.36–6.55)	0.5680	2.14 (0.27–16.83)	0.4682
ASA score, 1 or 2	-	-	1.00	-
ASA score, 3	0.14 (0.02–1.20)	0.0729	1.00	-
ASA score, 4 or 5	7.32 (0.83–64.43)	0.0729	26.45 (0.82–857.50)	0.0650

*RSCL* rapid source control laparotomy, *PRBC* packed red blood cells, *C* Celsius, *INR* international normalized ratio, *ASA* American Society of Anesthesiologists

^a^Unadjusted univariate analyses were used to test an independent variables’s predictive ability on in-hospital mortality

^b^Model covariates for mortality were chosen by full selection

^c^ *P*-value of <0.05 significant

Of the 162 non-RSCL emergent laparotomies, 27 (17 %) required unplanned re-explorations. Mortality in this unplanned take-back group was significantly higher than in the patients who did not require re-exploration (33.3 % vs 17.8 %; *p* = 0.04). Of the 33 deaths in the non-RSCL cohort, 27.3 % were patients necessitating unplanned re-explorations. Compared to the non-RSCL patients who did not undergo re-exploration, the unplanned take-back group had significantly higher rates of pre-operative severe sepsis / septic shock (44.4 % vs 11.9 %; *p* < 0.0001). Of all severe sepsis / septic shock patients in the non-RSCL group, 42.9 % (12) required re-exploration (*p* < 0.0001).

## Discussion

The negative downstream consequences of inflammation are well established in acute care surgery. In the trauma patient population, severe physiologic derangements, particularly the lethal triad (hypothermia, acidosis, and coagulopathy), guide management decisions [22]. Research into these postinjury systemic inflammatory states has established the importance, for example, of the damage control (DC) laparotomy for early definitive source control [1–6], and more recently the concept of DC resuscitation [23]. In critically ill patients, the substantial benefits of blunting the proinflammatory immune response has led to such key practices as early goal-directed therapy [24] and standardized treatment protocols for sepsis [25–27].

In EGS patients, upregulation of the systemic inflammatory response leads to a cascade of physiologic insults, and is a prime contributor to death, with mortality rates for severe sepsis / septic shock of over 40 % [14, 19]. However, unlike in trauma and critically ill patients, where physiologic derangements are aggressively acted on with specific corrective interventions, in EGS patients similar preoperative derangements are often recognized but may not be targeted with explicit restorative management techniques [28].

One solution to mitigating the negative downstream effects of physiologic insults seen preoperatively in EGS patients has been the application of DC techniques. Akin to the DC concept in trauma, it has been theorized that EGS patients needing operative intervention may benefit from an abbreviated laparotomy (i.e. rapid source control laparotomy; RSCL) with planned take-back. Such an operation is one of the first stages on a continuum of care that prioritizes the restoration of physiologic normality and homeostasis, and deemphasizes the importance of immediate organ repair and definitive anatomic reconstruction.

To guide the use of the staged RSCL in EGS patients, trauma’s lethal triad has been applied as the standard criteria for operative decision-making. The data to support this, however, is limited [29]. Furthermore, “expert opinion” about the timing of nontraumatic surgical emergencies has been published to guide surgical interventions in EGS patients [30]. To date, no data or empirical evidence has specifically defined a unique set of criteria on which to base the use of RSCL techniques to improve the poor survival rates of physiologically decompensated EGS patients.

Our results represent such data, and suggest that the acute physiologic indicators which help guide operative decisions in trauma patients may not confer a similar survival advantage to decompensated EGS patients undergoing an emergent laparotomy. In the 53 patients who underwent a staged RSCL based on the lethal triad, adjusted multivariable regression analysis shows that when used alone, no component of the lethal triad independently improved survival (Table 3). Therefore, contrary to current practices, application of the lethal triad does not appear to improve survival rates in EGS patients, and may possibly lead to overuse of RSCL with higher mortality rates (Table 2).

These data also illustrate that the indications for performing a staged RSCL in EGS patients should be founded on a set of unique preoperative physiologic parameters, coupled with inherent patient traits, which collectively define an EGS patient as physiologically decompensated (Tables 4 & 5). Our results suggest a profile of a patient who may realize a survival benefit from a RSCL: a patient with preoperative severe sepsis / septic shock, with an elevated lactate (≥3); acidosis (pH ≤ 7.25); elderly (≥70); male gender; and multiple comorbidities (≥3).

Patients with severe sepsis / septic shock had equally high unadjusted mortality rates in both cohorts: 53.6 % in the non-RSCL group and 60.0 % in the RSCL group (Table 2). In the risk-adjusted analyses for the non-RSCL group, presence of severe sepsis / septic shock was independently related to higher mortality rates, with an adjusted odds ratio for death of 12.3 (Table 4). In contrast, in the RSCL group, severe sepsis / septic shock was not independently predictive of increased risk of death (Table 5).

Collectively, our study suggests that emergency operative patients with severe sepsis / septic shock may benefit from a staged, abbreviated laparotomy, and may have an increased risk of death with an unstaged operative approach. Furthermore, in the non-RSCL cohort, 44.4 % of all patients who required unplanned re-explorations had severe sepsis / septic shock preoperatively, compared to only 11.9 % in the patients who were successfully unstaged. These data also document that nearly 50 % of patients with severe sepsis/septic shock undergoing laparotomy with primary fascial closure will require re-exploration (known as an on-demand re-laparotomy).

Similar patterns exist in the data for males and the elderly; these inherent patient traits independently and significantly increased the risk of mortality in emergent non-RSCL, but were not significantly related to mortality in the RSCL cohort (Tables 4 & 5). As such, men and those over the age of 70 may have poorer outcomes when they undergo unstaged, non-RSCL at the time of initial operation. Another inherent patient trait, multiple comorbidities, was independently associated with improved survival for both the non-RSCL and RSCL groups (Tables 4 & 5). Taken together, these data indicated that the elderly (≥70), males, and patients with multiple comorbidities (≥3) may see lower odds of mortality with RSCL emergent operations.

Acidosis (pH ≤ 7.25) and elevations in lactate (≥3) often occur in concert with each other, but presence of one does not necessarily mean presence of the other. We therefore modeled these variables independently from one another, as there was not a significant interaction between these variables in our analyses. In the RSCL group, elevated lactate independently improved chances of survival in patients undergoing abbreviated laparotomies; lactate was not significantly predictive of survival benefit in the non-RSCL cohort. Patients with acidosis (pH ≤ 7.25), on the other hand, have a very poor prognosis across all emergent operations (Table 2). Acidosis was the one variable associated with poor outcomes in both the non-RSCL and RSCL groups (Tables 4 & 5). Because odds of mortality go up significantly and independently with acidosis, and because survival is 50 % in acidotic patients, we have included it as an indication for RSCL.

The data further confirm that certain emergency patients will not benefit from RSCL, and in fact could be harmed by undergoing abbreviated operations. Specifically, patients without physiologic evidence of preoperative inflammation, as defined by the SIRS criteria, should not be considered candidates for RSLC. This cohort can, in most scenarios, safely undergo a non-staged operation; this is akin to the principle of on-demand re-laparotomy [17].

This study has limitations. First, our conclusions are drawn from retrospective data, and are thus constrained by the limitations and biases therein, including selection bias. Second, the definition of an emergency patient is a construct of the study, as well as the individual assessments of the attending surgeon and anesthesiologist. Therefore, generalizing to all emergency surgical patients may not be valid, as definitions of “emergency patient” are not standardized [31]. Thirdly, the data are from one institution, which is a tertiary medical center and large academic hospital; this may not be representative of a national sample from which to make conclusions. Fourth, our conclusions are drawn from a small sample size of 215 patients; we therefore lack the necessary power to show potentially significant differences. And finally, there may be other peri-operative risk factors and physiologic variables not identified here which more accurately predict the need for RSCL in EGS patients.

## Conclusions

In conclusion, when correctly applied, the staged RSCL may help to improve survival in decompensated EGS patients. The best candidates for staged RSCL are those patients with severe sepsis / septic shock, who are also male, over age 70 years old, with multiple comorbidities, and have an elevated lactate with acidosis. Use of the staged RSCL can also avoid unplanned re-explorations, which occurred in nearly 50 % patients with severe sepsis / septic shock who underwent primary fascial closure at the initial operation. Patients without evidence of preoperative inflammation may not benefit from staged RSCL. These results further reinforce the concept that EGS patients represent a unique patient-population, and that operative decision-making in these patients should be guided by EGS-specific preoperative and intraoperative physiologic metrics and variables.

## Abbreviations

ASA: American Society of Anesthesiologist Score
DC: Damage control
EGS: Emergency general surgery
ICU: Intensive Care Unit
NSQIP: National surgical quality improvement program
RSCL: Rapid source control laparotomy
SIRS: Systemic inflammatory response syndrome
